# Finite momentum Cooper pairing in three-dimensional topological insulator Josephson junctions

**DOI:** 10.1038/s41467-018-05993-w

**Published:** 2018-08-28

**Authors:** Angela Q. Chen, Moon Jip Park, Stephen T. Gill, Yiran Xiao, Dalmau Reig-i-Plessis, Gregory J. MacDougall, Matthew J. Gilbert, Nadya Mason

**Affiliations:** 1 Department of Physics and Frederick Seitz Materials Research Laboratory, University of Illinois, Urbana, 61801 IL United States; 2 Department of Electrical and Computer Engineering, University of Illinois, Urbana, 61801 IL USA

## Abstract

Unconventional superconductivity arising from the interplay between strong spin–orbit coupling and magnetism is an intensive area of research. One form of unconventional superconductivity arises when Cooper pairs subjected to a magnetic exchange coupling acquire a finite momentum. Here, we report on a signature of finite momentum Cooper pairing in the three-dimensional topological insulator Bi_2_Se_3_. We apply in-plane and out-of-plane magnetic fields to proximity-coupled Bi_2_Se_3_ and find that the in-plane field creates a spatially oscillating superconducting order parameter in the junction as evidenced by the emergence of an anomalous Fraunhofer pattern. We describe how the anomalous Fraunhofer patterns evolve for different device parameters, and we use this to understand the microscopic origin of the oscillating order parameter. The agreement between the experimental data and simulations shows that the finite momentum pairing originates from the coexistence of the Zeeman effect and Aharonov–Bohm flux.

## Abstract (web summary)

Unconventional superconductivity may emerge from the interplay between strong spin–orbit coupling and magnetism. Here, Chen et al. report an anomalous Fraunhofer pattern in three-dimensional topological insulator Bi_2_Se_3_ and attribute it as a signature of finite momentum Cooper pairing.

## Introduction

In the conventional Bardeen–Cooper–Schrieffer (BCS) theory of superconductivity, Cooper pairs form an isotropic condensate with a zero center-of-mass momentum^1^. However, introducing magnetism can change the stability of the BCS superconducting state, thereby destroying superconductivity or, as in unconventional superconductors, altering the pairing symmetry^2^. The potential for unconventional superconductivity at the confluence of magnetism and superconductivity has made it an area of great theoretical and experimental interest. One such example of an unconventional superconducting state is Fulde–Ferrell–Larkin–Ovchinnikov (FFLO) superconductivity, which was proposed as a way for maintaining superconductivity even beyond the critical Zeeman field^3,4^. Despite the intensive search for an FFLO superconductor in various types of materials such as heavy fermion compound CeCoIn_5_^5,6^ and BEDTTTF-based organic superconductors^7–9^, FFLO superconductivity still remains a controversial subject^2,10–12^.

To better hunt for unconventional superconductivity, there have been proposals for utilizing materials with strong spin–orbit interaction coupled to a conventional s-wave superconductor. This is predicted to stabilize an FFLO superconducting state: the spin–orbit coupling lifts the degeneracy in the Fermi surfaces of the material and introduces an anisotropy to the surfaces that makes it more amenable to a finite momentum phase^13–15^. In particular, time-reversal invariant topological insulators (TIs), whose surface states are massless Dirac fermions, are proposed to be an attractive candidate for unconventional superconductivity that carries finite momentum pairing^16^. To the best of our knowledge, experimental signatures of finite momentum Cooper pairs in TIs have mainly been sought after in the electron-doped regime of the two-dimensional (2D) TI HgTe quantum wells^17^, but the surface states of three-dimensional (3D) TIs also provide unique advantages to engineering finite momentum pairing. The Dirac cones on the surfaces are non-degenerate and have spin-momentum locking. As a consequence, the Fermi surface of the Dirac cone shifts uni-directionally under the application of an in-plane magnetic field to the surface, which can lead to an FFLO state^13,16^. Even though transport measurements in normal 3D TIs are often complicated by the presence of bulk carriers, there is experimental consensus that the metallic surface state dominates transport in a proximity-coupled TI even when the bulk is not depleted^18–22^.

To this end, we study the experimental signatures of Cooper pairs in a superconductor (S)-3D TI-S Josephson junction subjected to in-plane and out-of-plane magnetic fields. We probe the phase of Cooper pairs by generating Fraunhofer patterns with an out-of-plane field, and we find that adding an in-plane field distorts the Fraunhofer patterns by (1) transferring the intensity of superconductivity from the central Fraunhofer peak at *B*_*z*_ = 0 out to finite magnetic field values and (2) introducing asymmetries between positive and negative values of *B*_*z*_ in the Fraunhofer patterns. We show that the intensity transfer is suggestive of a spatially oscillating superconducting order parameter phase, which we call a finite momentum shift; we propose two potential origins for this finite momentum shift; and we demonstrate that asymmetries in the transport signal come from the sample geometry. Simulations show a close match between experimental data and finite momentum Cooper pair theory.

## Results

### Experimental set-up

**Fig. 1:**
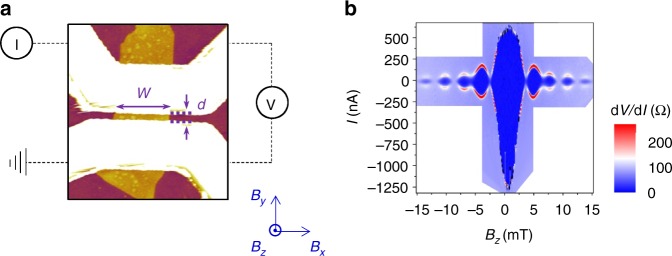
Measurement configuration and Device 1 Fraunhofer pattern. **a** AFM image of the S-TI-S Josephson junction: superconducting leads (white) on a Bi_2_Se_3_ flake (yellow) exfoliated onto a substrate (red). Measurement scheme and magnetic field configurations are also shown. **b** Conventional Fraunhofer pattern for device 1 (*t ~*9 nm, *d ~*140 nm, and *W ~*920 nm). The conventional pattern has a principal peak at *B*_*z*_  = 0

Our devices consist of Bi_2_Se_3_ flakes that are mechanically exfoliated from crystals with a bulk carrier density *n* ~ 5 × 10^17^ cm^−3^ (see Supplementary Note 1). Angle-resolved photoemission (ARPES) measurements have been made in a previous work on crystals similar to the ones used in our experiments, and they show that the Fermi energy is close to the bulk gap^23^. The flakes are contacted with two superconducting electrodes, forming a Josephson junction device, and the measured devices vary in flake thicknesses and junction dimensions (Table 1). A representative atomic force microscope (AFM) image is shown in Fig. 1a for device 1, which has flake thickness *t* ~9 nm, average junction width *W ~*860 nm, and electrode spacing of 140 nm. Because we utilize high in-plane fields to tune the behavior of the junction, we choose NbTi/NbTiN (*T*_c_*~*12.5 K and *H*_c2,in-plane_ > 9 T) as the superconducting material.

**Table 1:** Dimensions for devices 1–5

Device number	*t* (nm)	Average *W* (nm)	*d* (nm)	$\alpha = \frac{W_1}{W_2}$
1	9	860	140	1.07
2	11	1930	240	1.04
3	12	570	160	1.15
4	21	500	270	1.00
5	18	940	220	1.04

Out-of-plane magnetic field *B*_*z*_ is applied to the superconducting junction to generate a Fraunhofer pattern. In the absence of any in-plane fields, the devices exhibit a central peak with maximum critical current *I*_c_ and decaying side peaks, as is expected for a conventional Fraunhofer pattern. The nodes of our Fraunhofer pattern are at $B_z = \frac{{n{\mathrm{\Phi }}_0}}{{Wd}},$ where magnetic flux quantum ${\mathrm{\Phi }}_0 = \frac{h}{{2e}}$, *d* is the effective electrode spacing that takes into account flux focusing (see Supplementary Note 2), and *n* is an integer^24^. The Fraunhofer pattern of device 1, shown in Fig. 1b, is representative of our devices.

**Fig. 2:**
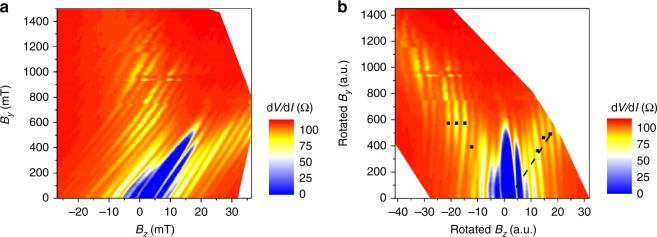
Device 1 Fraunhofer evolution. **a** Evolution of the Fraunhofer patterns for device 1. **b** Fraunhofer evolution for device 1 that has been rotated so that the lobe minima are vertical, making it easier to compare across samples. There is a side branch feature that develops as *B*_*y*_ is applied to the junction, which can be quantified as a line with slope *m* (dashed line). Black dots mark approximate locations of minimum resistance at different side lobes as a guide to the eye

When an in-plane field along the current direction *B*_*y*_ is introduced to the devices, the conventional Fraunhofer pattern is modulated to an anomalous Fraunhofer pattern: the Fraunhofer pattern is shifted along the *B*_*z*_ direction and the critical current of the side lobes increases as the critical current of the central lobe disappears. To measure the evolution of the Fraunhofer pattern as a function of *B*_*y*_, we apply a small AC excitation (with zero DC current) and measure the differential resistance d*V*/d*I* as a function of *B*_*z*_ and *B*_*y*_, where lower resistance corresponds to higher critical current, similar to in ref. ^17^. The evolution of the Fraunhofer pattern for device 1 is shown in Fig. 2a. As *B*_*y*_ is applied, the Fraunhofer pattern is shifted along *B*_*z*_. This is evident as an overall tilt of the 2D differential resistance map in Fig. 2a. Although a tilt could be caused by misalignment of the sample with respect to the *B*_*y*_–*B*_*z*_ plane, this type of misalignment would cause a similar shift in the Fraunhofer pattern when a field is applied in any in-plane direction. Because we do not see a corresponding shift when an in-plane field perpendicular to current direction *B*_*x*_ is applied, we can exclude sample misalignment as a cause for the shift of the Fraunhofer pattern.

Besides the shift to the Fraunhofer pattern, we also observe additional side branch features in the evolution of the Fraunhofer pattern: at *B*_*y*_ = 0, the intensity of superconductivity is maximum at the central lobe, but as *B*_*y*_ is increased, the intensity is transferred outwards to higher values of *B*_*z*_. The emergence of this anomalous side branch becomes more evident if the tilt in the 2D differential resistance map is removed by rotating the graph until the lobe minima are vertical, as shown in Fig. 2b. The approximate locations of the minima of the lifted side lobes are marked as a guide to the eye, and a slope for the side branch can be approximated, as illustrated by the dashed black line in Fig. 2b (see Supplementary Note 3). This unique Fraunhofer evolution is evidence of finite momentum pairing, as discussed below.

### Modeling Josephson junction with finite momentum pairing

To determine the origin of the evolution of the Fraunhofer pattern, we begin by considering the mesoscopic effects of magnetic fields *B*_*z*_ and *B*_*y*_ on the superconducting order parameter phase. For the following discussion we look primarily at the surface contribution because we find that the bulk order parameter decays much more rapidly than the surface contributions in the junction, which means the supercurrent is predominantly carried by the surface states (see Supplementary Notes 4 and 5). The phase modulation manifests itself as a spatially oscillating current distribution in the $\hat x$-direction ($I\left( x \right) = i_{\mathrm{c}}{\mathrm{sin}}( {\Delta \phi - \frac{{2eB_zxd}}{c}} )$)^24^. By summing up all the oscillating components of the current, the conventional Fraunhofer diffraction pattern arises, which can be derived from the following equation:

1 $$I_{\mathrm{c}} = i_{\mathrm{c}}W\left| {\frac{{\sin \left( {\frac{{{\mathrm{\pi \Phi }}}}{{{\mathrm{\Phi }}_0}}} \right)}}{{\frac{{{\mathrm{\pi \Phi }}}}{{{\mathrm{\Phi }}_0}}}}} \right|.$$

**Fig. 3:**
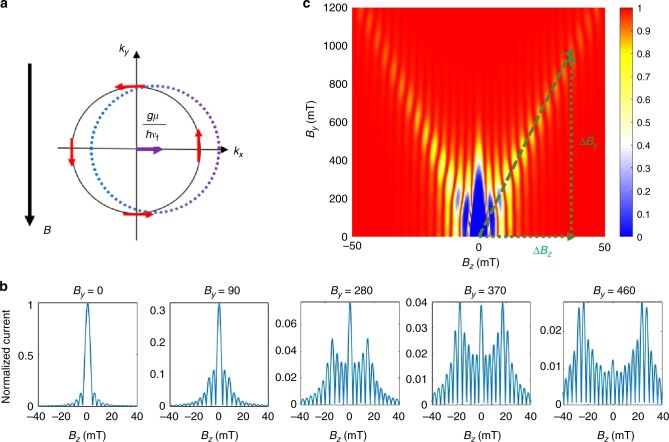
Finite momentum shift and simulation of trident pattern for ideal junction. **a** The shifted Fermi surface and spin texture (red arrows) of the TI due to a finite Zeeman effect. To form a spin singlet, Cooper pairs acquire a non-zero center of momentum, as indicated by the purple arrow. **b** Simulations of the Fraunhofer pattern for different values of *B*_*y*_. We find that the intensity of superconductivity is transferred outwards to higher values of *B*_*z*_ as *B*_*y*_ is increased. **c** Evolution of the Fraunhofer pattern for a symmetric Josephson junction due to finite momentum pairing. The differential resistance is calculated and normalized to 1. The slope of the side branch is indicated by the dashed green line

While the Fraunhofer pattern is generated by *B*_*z*_, the additional high in-plane magnetic field, *B*_*y*_, generates a Zeeman effect within the surface bands and adds magnetic flux inside the TI flake. When the in-plane Zeeman effect is present, the low-energy Hamiltonian of the TI surface can be written as

2 $$H_{{\mathrm{Dirac}}} = - \hbar v_{\mathrm{f}}\left( {k_x - \frac{{g\mu B_y}}{{\hbar v_{\mathrm{f}}}}} \right)\sigma _y + \hbar v_{\mathrm{f}}k_y\sigma _x,$$

where *v*_f_ is the Fermi velocity of the Dirac cone, *g* is the *g*-factor, and *μ* is the Bohr magneton^25^. By examining the Hamiltonian, we find that the location of the Dirac node is shifted from the Γ-point along the $\hat x$-direction by $\frac{{g\mu B_y}}{{\hbar v_{\mathrm f}}}$, resulting in a shift of the corresponding Fermi surface. Figure 3a shows a schematic of the shifted Fermi surface. As a result of this shift, when the electrons on the TI Fermi surface form spin singlet Cooper pairs, the Cooper pairs gain a finite center of mass momentum $\frac{{2g\mu B_y}}{{\hbar v_{\mathrm{f}}}}$. As a consequence, the superconducting order parameter at the end of each junction has a phase modulation in the $\hat x$-direction. The order parameter is given as ${\mathrm{\Delta }}_{{\mathrm{L}},{\mathrm{R}}} \approx {\mathrm{\Delta }}_0{\mathrm e}^{{\mathrm{i}}\frac{{2g\mu B_y}}{{\hbar v_{\mathrm{f}}}}x}$, where $\frac{{2B_yxg\mu }}{{\hbar v_{\mathrm{f}}}}$ is the phase modulation due to a finite momentum shift. The finite momentum of the Cooper pair under these conditions is similar in nature to FFLO states^2,3^. Unlike for the Fermi surface, the bulk energy eigenvalue is degenerate. The Zeeman effect will therefore only lift the spin degeneracy rather than shifting the bulk bands, so the bulk does not acquire a phase from the Zeeman effect (see the Methods section).

Besides the Zeeman effect contribution to the order parameter, there is also a contribution from the finite flux that is inserted along the in-plane, or $\hat y$, direction of the flake. This magnetic flux from *B*_*y*_ results in a phase modulation given by an Aharonov–Bohm phase $\frac{{2{\mathrm{\pi }}}}{{\phi _0}}\mathop {\int }\limits_{r_1}^{r_2} {\mathbf{A}} \cdot {\mathrm {d}}l$^26^. For a bulk electron, the phase will be determined by its trajectory inside the flake, and we find that the bulk electrons will acquire a negligible net phase modulation (see the Methods section and Supplementary Note 6). For a surface electron, on the other hand, the phase modulation is given as $\frac{{{{\pi B}}_{{y}}xt}}{{{\mathrm{\Phi }}_0}}$. As a result, we see that there are two finite momentum shift contributions to the surface order parameter of the 3D TI: the Zeeman effect and the Aharonov–Bohm effect, which we call the Zeeman modulation effect (ZME) and flux modulation effect (FME), respectively.

By summing up the three relevant contributions to the phase—the out-of-plane magnetic field *B*_*z*_, the ZME, and the FME—we get the total phase difference between the two junctions:

3 $${\phi _1\left( {x_1} \right) - \phi _2\left( {x_2} \right) = \frac{{2{\mathrm{\pi }}B_zd\left( {x_1 + x_2} \right)}}{{2{\mathrm{\Phi }}_0}} + \frac{{2B_y\left( {x_1 - x_2} \right)g\mu }}{{\hbar v_{\mathrm{f}}}} + \frac{{{\mathrm{\pi }}B_y\left( {x_1 - x_2} \right)t}}{{{\mathrm{\Phi }}_0}}}.$$

Here, *ϕ*_1_(*x*_1_) and *ϕ*_2_(*x*_2_) are the phases of the order parameters of superconductor 1 and 2 at the coordinates *x*_1_ and *x*_2_, respectively, along the width of the junction. Based on the phase difference between the two leads, we can model the total transport current along the $\hat y$-direction in the Josephson junction using quasi-classical methods^17,27^. This analysis is equivalent to summing up all possible quasi-classical trajectories of electron transport, so the total transport current is

4 $${I\left( {\phi ,B_y,B_z} \right) = {\int \nolimits_{ - \frac{{W_1}}{2}}^{\frac{{W_1}}{2}}} {\int \nolimits_{ - \frac{{W_2}}{2}}^{\frac{{W_2}}{2}}} {\mathrm {d}}x_1{\mathrm {{d}}x_2\frac{1}{{d}^2 + \left( {x_1 - x_2} \right)^2}}\,{\mathrm{sin}}\left( {\Delta \phi + \phi _1\left( {x_1} \right) - \phi _2(x_2)} \right){\mathrm{,}}}$$

where *W*_1(2)_ is the width of the superconducting lead 1(2), Δ*ϕ* is the overall phase difference between the superconductors, and *d* is the distance between the two superconductors. Using Eq. (4), we can calculate the critical current $I_{\mathrm{c}}({B_y,B_z} ) = \max _{\phi} I({\phi ,B_y,B_z})$ as a function of *B*_*z*_ and *B*_*y*_ to derive the evolution of the Fraunhofer pattern.

Figure 3b shows simulations of the Fraunhofer pattern for various values of *B*_*y*_ calculated using Eq. (3) and illustrates how the intensity of superconductivity is transferred from the central peak out to the side peaks as *B*_*y*_ is increased. This transfer of intensity is proportional to the momentum shift of the Cooper pair. In terms of the differential resistance as a function of *B*_*z*_ and *B*_*y*_, the transfer of the superconducting intensity can be seen as the formation of two side branches (formed by evolving side peaks) in the differential resistance map with slope *m*. These side branches are visible in the simulation for a symmetric Josephson junction in Fig. 3c and in the data for device 1 (Fig. 2b). The agreement between the simulation and the experimentally observed pattern indicates that the formation of the side branches is a result of the transfer of superconducting intensity due to the additional phase modulation generated by *B*_*y*_. This feature is known to be a key signature of finite momentum pairing and distinguishes the system from typical BCS superconductivity^16,28,29^.

Additionally, the slope *m* of the side branches reflects the relative contributions of finite momentum pairing due to ZME and FME as a function of *B*_*y*_. In the simulations, the slope is defined by a line between the origin and the *n*th side lobe as *n* becomes large (green dashed line in Fig. 3c). By calculating the integral in Eq. (4), the slope of the side branches is estimated as

5 $$m = \frac{{{\mathrm{\Delta }}B_y}}{{{\mathrm{\Delta }}B_z}} = \frac{{{\mathrm{\pi }}d/{\mathrm{\Phi }}_0}}{{\frac{{2g\mu }}{{\hbar v_{\mathrm{f}}}} + \frac{{2{\mathrm{\pi }}t}}{{{\mathrm{\Phi }}_0}}}}.$$

In Eq. (5), the first and the second terms in the denominator are the contributions of the ZME and the FME to the slope, respectively. Because of the inverse relation, larger slopes reflect smaller ZME and FME contributions. Looking at the FME and ZME contributions separately, we can see that the FME contribution is proportional to the thickness of the flake *t* since the flux through *B*_*y*_ will increase as the thickness increases. The ZME, on the other hand, is proportional to the intrinsic material parameters of the TI, $\frac{g}{{v_{\mathrm{f}}}}$, rather than on an external parameter, such as the thickness.

**Fig. 4:**
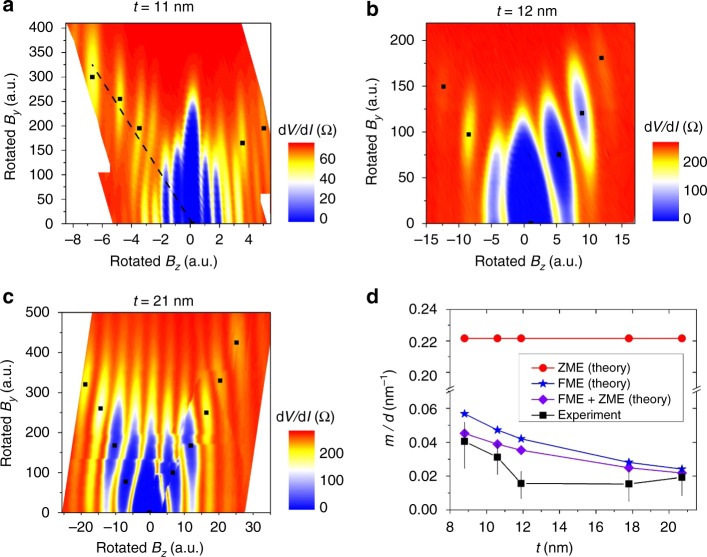
Fraunhofer evolution for devices with thicker flakes. **a**–**c** Fraunhofer evolution for devices 2–4, which have different flake thicknesses. For comparison, the data were rotated so that the lobe minima are vertical for better comparison and approximate minima are marked with black dots. The side branch slope is illustrated for device 2 in **a**. **d** The relation between the slope of the side branch *m* (normalized by effective electrode distance *d*) and thickness *t* of the TI flake. Experimental data (with error bars for deviations in extracted slopes) are compared with simulations for each device using a finite momentum pair model. The theory and data matches best for a model that takes into account both ZME and FME. *v*_f_ = 5 × 10^5^ m s^−1^, *g* = 19 are used in the simulations^31,32^

We examine the slope dependence on TI thickness across multiple samples, where devices 2–4 are shown in Fig. 4a–c, respectively, with approximate minima marked and the 2D differential resistance maps rotated so that the lobe minima are vertical for ease of comparison. The slope *m* for each device is extracted from the minima (see Supplementary Note 3) and is illustrated for device 2 by the dashed line in Fig. 4a. To compare the experimental data with theory, we also calculate *m* for each device based on *t* and *d* using Eq. (5). Figure 4d shows the dependence of *m* (normalized by an effective *d* that takes into account flux focusing effects) on thickness, where *m* is extracted from the data (black) and calculated using theoretical predictions for finite momentum pairing due to ZME alone (red), FME alone (blue), and ZME and FME together (purple).

It is clear that the dominant contribution to the finite momentum shift comes from the FME. In the thickest sample, in particular, the FME closely predicts the slope in the experiment since more flux is enclosed in the flake and the orbital effect therefore has a more significant contribution. However, there are some deviations between the data and the FME curves. First, some of the normalized slopes do not follow the monotonically decreasing trend predicted by the FME theory. The deviations are caused by variations in the sample: in real samples, there are variations in chemical potentials, which would result in the $\frac{g}{{v_{\mathrm{f}}}}$ ratio changing from sample to sample, and fabrication imperfections, which would result in distortions in the transport signal. Nevertheless, the overall decreasing trend in the experimental slopes mirrors the decreasing trend predicted by the FME model.

We also find that the FME is not enough to predict the observed slope from the experiment on its own even when the error bars of slope calculation are taken into account. In fact, the ZME contribution needs to be considered for the theory to more closely match the data, which means that the finite momentum pairing due to the shifted Dirac cones is non-negligible. We calculate a curve that takes into account an additional ZME value (purple) that demonstrates that by introducing the ZME contribution to the theory, the theory curve is shifted closer to the experimental data. Therefore, our data are generally better explained by the coexistence of both FME and the unconventional ZME, which is more closely related to the FFLO state.

We also looked at the potential phase contribution from inserting a flux through the superconducting leads. In the supplementary section of a previous work^17^, it was found that a flux through the electrode seemed to contribute to the phase modulation in the system. However, the normalized slopes are not correlated with the thickness of the device leads in our experiment. This suggests that the relevant flux for the finite momentum pairing is the flux through the flake.

### Effect of Josephson junction asymmetries on the evolution of the Fraunhofer pattern

In addition to simulating the Fraunhofer evolution as *B*_*y*_ is applied to an ideal junction, we also consider the effect of asymmetries in the junction geometry and on the evolution of the Fraunhofer pattern. Due to the fact that typical sample fabrication can result in imperfect device configurations and flux focusing effects, it is important to understand what happens to the Fraunhofer pattern as the devices deviate from the ideal junction. For example, as reported in ref. ^30^, asymmetric features between positive and negative values of *B*_*z*_ often appear in Fraunhofer patterns and can be attributed to a combination of device-dependent factors such as disorder and the microscopic structure of the device. We consider some sources of asymmetries in the device configuration to model the effect of these asymmetries on the transport signal.

**Fig. 5:**
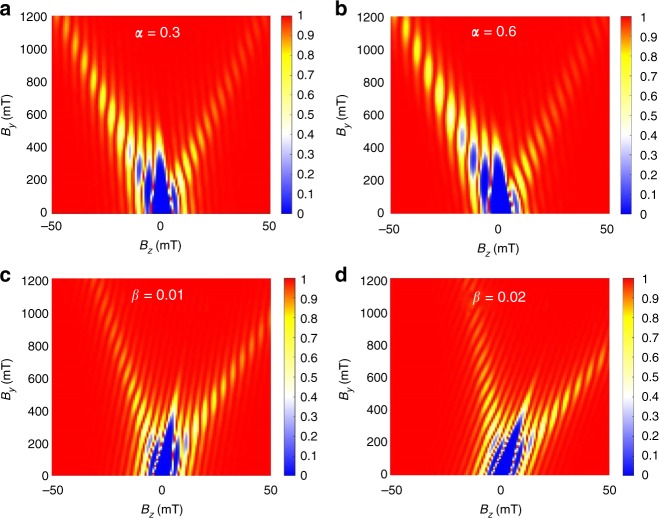
Simulation of effect of junction asymmetry and field inhomogeneity. The width and field asymmetry dependence of the Fraunhofer pattern. **a**, **b** When a width asymmetry factor *α* is added to the model, we find that the signal becomes asymmetric between positive and negative *B*_*z*_. Here, the amplitude of the left side lobes increases relative to the amplitude of the right side lobes. We used the values *α* = 0.3, 0.6 respectively. **c**, **d** When the asymmetry factor *β* is introduced to the model, the Fraunhofer patterns are shifted along *B*_*z*_, which can be seen as a tilt introduced to the 2D differential resistance map. We used *β* = 0.01, 0.02 respectively

One form of geometric asymmetry that arises in a Josephson junction is the asymmetry in the width of the two superconducting leads *W*_1_ and *W*_2_. To model this effect, we introduce the width asymmetry factor *α*, which is the ratio of the two superconducting lead widths and satisfies *W*_1 _= *αW*_2_ in Eq. (4). Figure 5a, b shows how the Fraunhofer evolution changes as we increase the asymmetry between *W*_1_ and *W*_2_ by increasing *α*. Because finite *α* breaks the symmetry of $I({\phi ,B_y,B_z})$ upon reversing the sign of *B*_*z*_ in Eq. (4), we find that for increasing *α*, the amplitude of the left and right side branches becomes more asymmetric. Another form of asymmetry, as quantified by flux asymmetry factor *β*, comes from the flux focusing effect^30^. Due to the screening of the magnetic field inside the superconductor, magnetic field *B*_*y*_ may bend and cause contributions to *B*_*z*_. Since we apply large *B*_*y*_ compared to *B*_*z*_, a very small bending of *B*_*y*_ can cause a large tilt in the Fraunhofer pattern (see Supplementary Information). We model this effect by replacing *B*_*z*_ with (*B*_*z*_−*βB*_*y*_) in Eq. (3), which generates the tilt seen in Fig. 5c, d.

**Fig. 6:**
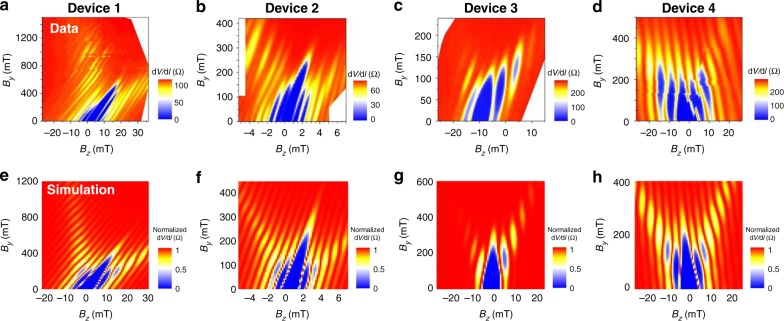
Evolution of the Fraunhofer pattern across different devices. Comparison of the evolution of the Fraunhofer pattern when *B*_*y*_ is applied to devices 1–4. The experimental data (**a**–**d**) agrees well with the normalized differential resistance of the simulations (**e**–**h**) that are based on a finite momentum shift model and take into account width asymmetries delineated in Table 1. An additional tilt is added to the simulations for better comparison with the data. (A possible origin for the tilt is a flux asymmetry, which is difficult to quantify.) In all the devices, side branches with slope *m* appear as an in-plane field *B*_*y*_ is added. The appearance of these side branches is indicative of interference in the phase modulation due to *B*_*z*_ and *B*_*y*_

After understanding possible origins for anomalous features in the evolution of the Fraunhofer pattern, we can now compare our experimental data with simulations that take into account asymmetry factors. The results are shown in Fig. 6 (see Supplementary Note 8 for the detailed numerical methods). The width asymmetry factor *α* is extracted from scanning electron microscope images of the devices (summarized in Table 1). Due to the difficulty of quantifying the magnitude of the flux asymmetry, we add in an artificial *β* factor to simulations that generates a tilt that best matches with the data for better comparison. As discussed earlier, the overall structure and in-plane field dependence of the Fraunhofer pattern is determined by the ZME, the FME, and the geometry of the junction. However, we find that by also incorporating the sample width asymmetries into the finite momentum pairing model and adding an artificial tilt in the data to take into account flux focusing effects, the theoretical prediction and the experiment agree very well.

## Discussion

In conclusion, we observe an anomalous Fraunhofer pattern that is indicative of the presence of finite momentum pairing in 3D TI Josephson junctions that are subjected to in-plane magnetic fields. We identify the two microscopic origins of the finite momentum pairing to be the ZME and the FME. By comparing the slope of the side branches in the anomalous Fraunhofer pattern with the theoretical predictions, we conclude that the measured slope can only be explained by the coexistence of both the ZME and the FME. In particular, the ZME—the contribution associated with the FFLO phase—becomes significant when there is less phase accumulation due to enclosed flux, which occurs for thinner samples. We believe that this is a promising start for the hunt for unconventional superconductivity in a proximity-coupled 3D TI in the presence of in-plane fields, but further work can be done to mitigate the effect of the FME. For example, besides finding thinner flakes, the ZME can be enhanced by increasing *g/v*_f_, which can be done by tuning the TI flake closer to the Dirac point. Furthermore, as demonstrated by our work and others, a continued understanding of the effect of non-ideal junctions is conducive to identifying anomalous asymmetric signatures, like the Fraunhofer asymmetry across *B*_*z*_, in transport signals.

## Methods

### Experimental techniques

3D TI flakes were mechanically exfoliated from bulk Bi_2_Se_3_ crystals onto Si/SiO_2_ substrates, and thicknesses were identified using atomic force microscopy. After identifying suitable flakes, superconducting electrodes were defined using standard ebeam lithography techniques. Forty to 65 nm of NbTi/NbTiN was then sputtered onto the device using an rf source following a brief ion mill to clean off the surface. Devices were then wire-bonded and measured in a dry dilution unit that reaches a base temperature of *T* = 25 mK and has a three-axis vector magnet.

### Numerical methods

After calculating the Josephson current using Eq. (4), we numerically generate Fraunhofer patterns as a function of *B*_*y*_ for each device. We find that a non-zero *B*_*y*_ transfers the intensity of superconductivity from *B*_*z*_ = 0 out to higher values of *B*_*z*_. To illustrate this feature, we present various slices of the Fraunhofer pattern as a function of *B*_*y*_ in Supplementary Figure 6. Once we derive a set of Fraunhofer patterns as a function of *B*_*y*_, we can draw the surface contour plot of the critical current as a function of both *B*_*y*_ and *B*_*z*_ and map the critical current map to a differential resistance map with an effective thermal noise, as detailed in Supplementary Note 7.

### Calculation of bulk and surface contributions to the ZME

In this section, we determine how the Zeeman effect affects surface and bulk states by calculating the normal propagator of the surface and the bulk of the TI. We first solve for the surface propagator. The Hamiltonian of the TI surface with a finite in-plane Zeeman term is given as

$$H_{{\mathrm{surf}}} = \hbar v_{\mathrm{f}}\left( { - \left( {k_x - \alpha } \right)\sigma _y + k_y\sigma _x} \right) - E_{\mathrm{f}},$$

where ${\mathrm{\alpha }} = \frac{{g\mu B}}{{\hbar v_{\mathrm{f}}}}$ and *E*_f_ is the Fermi energy. The energy of the above Hamiltonian is given as

$$\begin{array}{*{20}{c}} {E_{{\mathrm{surf}},\hskip -1pt \pm } = - E_{\mathrm{f}} \pm \hbar v_{\mathrm{f}}\sqrt {\left( {k_x - \alpha } \right)^2 + k_y^2} } \end{array}$$

with eigenstates

$$\left| {{\mathrm{\lambda }}, \pm } \right\rangle = \left( {\begin{array}{*{20}{c}} {{\mathrm {e}}^{ - {\mathrm{i}}\phi }} \\ { \pm 1} \end{array}} \right),$$

where $${\mathrm{e}}^{ - {\mathrm{i}}\phi } = \cos \left( \phi \right) - {\mathrm{i}}\;{\mathrm{sin}}(\phi ) = \frac{{k_y}}{{\sqrt {\left( {k_x - \alpha } \right)^2 + k_y^2} }} + {\mathrm{i}}\frac{{\left( {k_x - \alpha } \right)}}{{\sqrt {\left( {k_x - \alpha } \right)^2 + k_y^2} }}.$$

After deriving the eigenstate and the energies and taking just the positive eigenstate (since we assume the chemical potential is near the conduction band), we can use these results to solve for the surface propagator, *g*_surf_(*E*,*r*):

$$g_{{\mathrm{surf}}}\left( {E,{\mathbf{r}}} \right) = \frac{{k_{\mathrm {f}}}}{{\left( {2{\mathrm{\pi }}} \right)^2\hbar v_{\mathrm{f}}}}{\mathrm {e}}^{{\mathrm{i}}\alpha x}\mathop {\int }\limits_0^{2{\mathrm{\pi }}} {\mathrm {d}}\theta \,{\mathrm {e}}^{{\mathrm{i}}k_{\mathrm{f}}r{\mathrm{cos}}\left( {\theta _{{{k}}} - \theta _{{{r}}}} \right)}\left[ {\begin{array}{*{20}{c}} 1 & {{\mathrm {e}}^{ - {\mathrm{i}}\theta _{{{k}}}}} \\ {{\mathrm {e}}^{{\mathrm{i}}\theta _{{{k}}}}} & 1 \end{array}} \right],$$

where $k_{{\mathrm{f}}, \pm } = \frac{{E_{\mathrm{f}} + E}}{{\hbar v_{\mathrm{f}}}}$ is the Fermi wave vector. The above integration is the Bessel function of the first kind, *J*_0_:

$$g_{{\mathrm{surf}}}\left( {E,{\mathbf{r}}} \right) = \frac{{k_{\mathrm{f}}}}{{\left( {2{\mathrm{\pi }}} \right)^2\hbar v_{\mathrm{f}}}}{\mathrm {e}}^{{\mathrm{i}}\alpha x}\left[ {\begin{array}{*{20}{c}} {2{\mathrm{\pi }}J_0\left( {k_{\mathrm{f}}r} \right)} & {2{\mathrm{\pi i}}{\mathrm {e}}^{ - {\mathrm i}\theta _{{r}}}J_1\left( {k_{\mathrm{f}}r} \right)} \\ {2{\mathrm{\pi i}}{\mathrm {e}}^{{\mathrm{i}}\theta _{{k}}}J_1\left( {k_{\mathrm{f}}r} \right)} & {2{\mathrm{\pi }}J_0\left( {k_{\mathrm{f}}r} \right)} \end{array}} \right].$$

In the canonical quasi-classical approximation, we assume that $k_{\mathrm{f}}\,r \gg 1$, so the Bessel functions can be asymptotically approximated as

$$\begin{array}{ccccc}\\ g_{{\mathrm{surf}}}\left( {E,{{\mathbf{r}}}} \right) = \frac{{k_{\mathrm{f}}}}{{2{\mathrm{\pi }}\hbar v_{\mathrm{f}}}}{{\mathrm{e}}}^{{\mathrm{i}}\alpha x}\sqrt {\frac{2}{{{\mathrm{\pi }}k_{\mathrm{f}}r}}} \left[ {\begin{array}{*{20}{c}} {\cos \left( {k_{\mathrm{f}}r - \frac{{\mathrm{\pi }}}{4}} \right)} & {{\mathrm{i}}{\mathrm {e}}^{ - {\mathrm{i}}\theta _{{r}}}\cos \left( {k_{\mathrm{f}}r - \frac{{3{\mathrm{\pi }}}}{4}} \right)} \\ {{{\mathrm{i}}}{\mathrm {e}}^{{\mathrm\it{i}}\theta _{{r}}}\cos \left( {k_{\mathrm{f}}r - \frac{{3{\mathrm{\pi }}}}{4}} \right)} & {\cos \left( {k_{\mathrm{f}}r - \frac{{\mathrm{\pi }}}{4}} \right)} \end{array}} \right]\\ \\ \hskip 45pt= \frac{{k_{\mathrm{f}}^{\frac{1}{2}}}}{{\left( {2{\mathrm{\pi }}} \right)^{\frac{3}{2}}\hbar v_{\mathrm{f}}r^{\frac{1}{2}}}}{\mathrm {e}}^{{\mathrm{i}}\alpha x}\left[ {\begin{array}{*{20}{c}} {2\cos \left( {k_{\mathrm{f}}r - \frac{{\mathrm{\pi }}}{4}} \right)} & {2{\mathrm{i}}{\mathrm {e}}^{ - {\mathrm{i}}\theta _{\mathbf{r}}}\cos \left( {k_{\mathrm{f}}r - \frac{{3{\mathrm{\pi }}}}{4}} \right)} \\ {2{\mathrm{i}}{\mathrm {e}}^{{\mathrm{i}}\theta _{{r}}}\cos \left( {k_{\mathrm{f}}r - \frac{{3{\mathrm{\pi }}}}{4}} \right)} & {2\cos \left( {k_{\mathrm{f}}r - \frac{{\mathrm{\pi }}}{4}} \right)} \end{array}} \right].\\ \end{array}$$

This is the final expression for the surface propagator, where the e^i*ax*^ term represents oscillations in the surface propagator due to the Zeeman effect.

We now solve for the bulk propagator. To do so, we follow a similar procedure to the one used to solve for the surface propagator. Using Supplementary Eq. (2), the bulk Hamiltonian of the TI with the Zeeman effect can be written as

$$H_{{\mathrm{bulk}}} = - E_{\mathrm{f}} + m{\mathrm{\Gamma }}_0 + \hbar v_{\mathrm{f}}k \cdot \Gamma + \beta \tau _0\sigma _2,$$

where *β* = *gμB*. The bulk energy eigenvalue is given as

6 $$E_{{\mathrm{bulk}}} = - E_{\mathrm{f}} \pm \sqrt {k_x^2 + \left( {\sqrt {m^2 + k_y^2 + k_z^2} \pm \beta } \right)^2} .$$

It is important to note that Eq. (6) differs from the surface energy eigenvalue because, in the bulk, the Zeeman effect does not shift the location of the Fermi surface but rather only lifts the degeneracy. As a consequence, we will see that the ZME is absent from the bulk band contribution.

We again solve for the normal propagator

$$g_{{\mathrm{bulk}}}\left( {E,{\mathbf{r}}} \right) 	= \frac{1}{{\left( {2{\mathrm{\pi }}} \right)^3}}{\int} {d^3k} e^{{\mathrm{i}}{\mathbf{k}} \cdot {\mathbf{r}}}\mathop {\sum }\limits_\lambda \left| \lambda \right\rangle \left\langle \lambda \right|\delta \left( {E - E_\lambda \left( {\mathbf{k}} \right)} \right)\\ 	= \frac{1}{{\left( {2{\mathrm{\pi }}} \right)^3}} {\int \nolimits_0^\infty} k^2{\mathrm {d}}k {\int \nolimits_0^{\mathrm{\pi }} }{\mathrm{sin}}(\theta ){\mathrm {d}}\theta {\int \nolimits_0^{2{\mathrm{\pi }}}} {\mathrm {d}}\phi {\mathrm {e}}^{{\mathrm{i}}kr{\mathrm{cos}}\left( \theta \right)}\mathop {\sum }\limits_\lambda \left| \lambda \right\rangle \left\langle \lambda \right|\delta \left( {E - E_\lambda \left( k \right)} \right).$$

The eigenstate of the above Hamiltonian can be explicitly calculated by assuming $\left| k \right| \ll m$. In this case, we can focus on just the conduction band to simplify the expression:

$$g_{{\mathrm{bulk}}}\left( {E,{\mathbf{r}}} \right) 	= \frac{1}{{\left( {2{\mathrm{\pi }}} \right)^3}}\mathop {\sum }\limits_\lambda k_{\mathrm{f}}^2{\int \nolimits_0^{\mathrm{\pi }}} {\mathrm{sin}}(\theta ){\mathrm {d}}\theta {\int \nolimits_0^{2{\mathrm{\pi }}}} {\mathrm {d}}\phi {\mathrm {e}}^{{\mathrm{i}}k_{\mathrm{f}}r{\mathrm{cos}}(\theta )}\left| \lambda \right\rangle \left\langle \lambda \right|\\ 	= \frac{1}{{\left( {2{\mathrm{\pi }}} \right)^3}}k_{\mathrm{f}}^2{\int \nolimits_{ - 1}^1} {\mathrm{d}}c{\int \nolimits_0^{2{\mathrm{\pi }}} }{\mathrm {d}}\phi {\mathrm {e}}^{{\mathrm{i}}k_{\mathrm{f}}rc} = \frac{{k_{\mathrm{f}}^2}}{{\left( {2{\mathrm{\pi }}} \right)^2}}\left( {\frac{1}{{{\mathrm{i}}k_{\mathrm{f}}r}}\left( {{\mathrm {e}}^{{\mathrm{i}}k_{\mathrm{f}}r} - {\mathrm {e}}^{ - {\mathrm{i}}k_{\mathrm{f}}r}} \right)} \right)\\ 	= \frac{{k_{\mathrm{f}}}}{{\left( {2{\mathrm{\pi }}} \right)^2\hbar v_{\mathrm{f}}r}}2\sin \left( {k_{\mathrm{f}}r} \right).$$

Because the bulk band is initially degenerate and does not shift in one direction in momentum space, the bulk propagator does not have oscillations from the ZME.

### Calculation of surface and bulk quasiparticles of the Aharonov–Bohm phase

In addition to the Zeeman effect, the electrons gain additional phases due to the FME. In this section, we calculate the phase accumulated by electrons in a magnetic field as they acquire an Aharonov–Bohm phase. The vector potential of the in-plane magnetic field can be written as ${\mathbf{A}} = (B_y(z - \frac{t}{2}),0,0)$. Under this gauge, the surface electrons, which travel along the outmost trajectory of the TI, gain the conventional Aharonov–Bohm phase. More specifically, the electrons at the top surface, *z* = *t*, gain momenta opposite from the bottom surface, *z* = 0, which is consistent with a current circulating around the surface loop. When the surface electron classically travels from *x*_1_ to *x*_2_, the phase is given as

$$\phi \left( {x_2} \right) - \phi \left( {x_1} \right) 	= 2{\mathrm{\pi }} \times \frac{{{\mathrm{total}}\;{\mathrm{flux}}}}{{\phi _0}} \times \frac{{{\mathrm{travel}}\;{\mathrm{distance}}}}{{{\mathrm{circumference}}}} \\ 	= 2{\mathrm{\pi }}\frac{{tWB_y}}{{\phi _0}}\frac{{\left( {x_2 - x_1} \right)}}{{2W + 2t}}\sim \frac{{{\mathrm{\pi }}tB_y}}{{\phi _0}}\left( {x_2 - x_1} \right).$$

Unlike the surface electrons, the set of classical trajectories of the bulk electrons can be characterized by the number of reflections off the bottom surface (Supplementary Figure 4). Similarly the Green function can be decomposed as follows:

$$F\left( {{\mathrm{i}}\omega ,{\mathrm{r}}} \right) = F_0\left( {{\mathrm{i}}\omega ,{\mathrm{r}}} \right) + F_1\left( {{\mathrm{i}}\omega ,{\mathrm{r}}} \right) + \ldots ,$$

where *F*_*n*_ represent the normal propagators that consist of the trajectories having *n* reflections off the bottom surface. When the travel distance is longer than the magnetic length $l_B = \left( {\frac{{\hbar c}}{{eB}}} \right)^{1/2}$, the Green function gains the Aharonov–Bohm phase, $\frac{{2\pi }}{{\phi _0}}\mathop {\int }\limits_{r_1}^{r_2} \vec A \cdot {\mathrm {d}}l$, where the integral is taken over the chord connecting *r*_1_ and *r*_2_. There are two distinct Aharonov–Bohm phases that come from the bulk quasiparticle trajectories: (1) a phase due to a trajectory with no reflections so that the quasiparticle is transferred directly from one superconducting contact into the other and (2) a phase due to a trajectory that reflects off of the bottom surface more than once before being transferred into the other contact. If there are no reflections, the bulk quasiparticle will follow the same trajectory as the surface quasiparticles. In this case, the bulk electron should gain the same phase as the surface electron:

$$\phi \left( {x_2} \right) - \phi \left( {x_1} \right) = \frac{{2{\mathrm{\pi }}}}{{\phi _0}}{\int} {{\mathbf{A}} \cdot {\mathrm {d}}l} = \frac{{2{\mathrm{\pi }}B_y}}{{\phi _0}}\mathop {\int }\limits_{x_1}^{x_2} \left( {z\left( x \right) - \frac{t}{2}} \right){\mathrm {d}}x = \frac{{{\mathrm{\pi }}tB_y}}{{\phi _0}}\left( {x_2 - x_1} \right).$$

If the bulk quasiparticle is reflected from the bottom surface at least one time, it will follow a continuous path from *z* *=* 0 to *z* *=* *t* and the bottom half will have the opposite modulation from the top half of the flake. In this case, the net phase cancels each other out:

$$\phi \left( {x_2} \right) - \phi \left( {x_1} \right) = \frac{{2{\mathrm{\pi }}}}{{\phi _0}}{\int} {{\mathbf{A}} \cdot {\mathrm {d}}l} = \frac{{2{\mathrm{\pi }}}}{{\phi _0}}\mathop {\int }\limits_0^t \left( {z\left( x \right) - \frac{t}{2}} \right) = 0.$$

As a result, bulk electrons with reflection-less trajectories gain the same FME effect as surface electrons while the bulk electrons with reflected trajectories do not experience the FME and only contribute to the conventional Fraunhofer pattern.

### Data availability

The data that support the findings of this study are available from the corresponding author on reasonable request.

## Electronic supplementary material

Supplementary Information
