# Supplementary material for: Finite momentum Cooper pairing in three-dimensional topological insulator Josephson junctions
Source: Nat Commun. 2018 Aug 28;9:3478. doi: 10.1038/s41467-018-05993-w (PMC6113236; doi:10.1038/s41467-018-05993-w)
Supplement: Supplementary file 1 — Supplementary Information [file 41467_2018_5993_MOESM1_ESM.pdf]

Supplementary Figures

Supplementary Figure 1

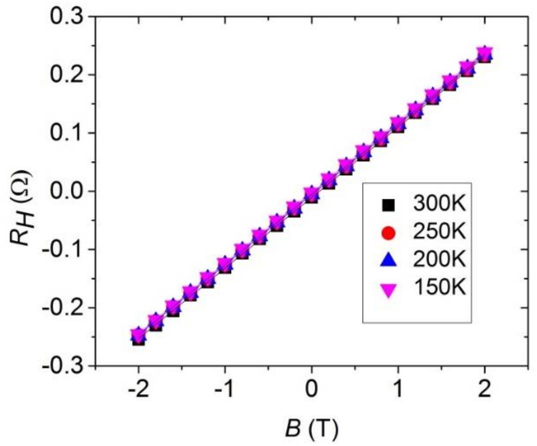

The bulk carrier density is extracted from the Hall data taken on Bi_2Se_3 crystals used to make the devices.

Supplementary Figure 2

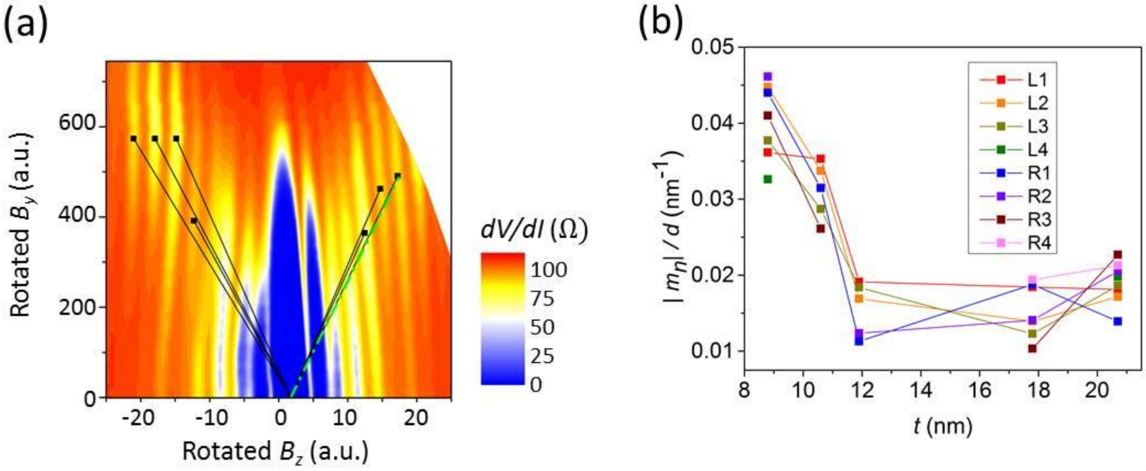

(**a**) Lines to the minima of each lifted side lobe have slope $|m_n|$ and are shown (black lines) for device 1. The average of these slopes, m, is also shown (dashed green line) and corresponds to the slope value used in the main text. (**b**) Normalized slopes $|m_n|$ are extracted for each device and plotted as a function of thickness t. Each color represents the slope to a different side lobe.

Supplementary Figure 3

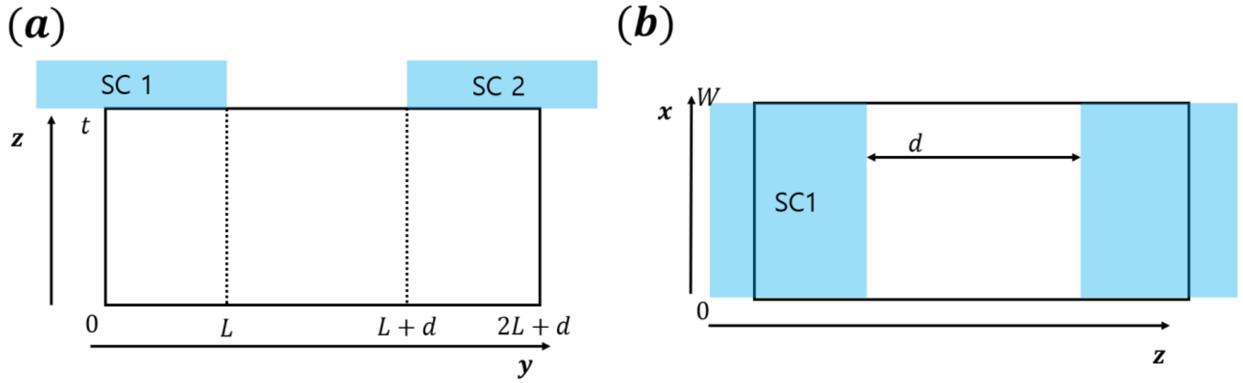

Schematic figure of the simulation. Blue(White) rectangles represent the superconductors(TI). The superconductors are placed on the top of the topological insulator. This is a valid assumption since the side area of the topological insulator is smaller compared to the top surface. (**a**) side view (**b**) top view.

Supplementary Figure 4

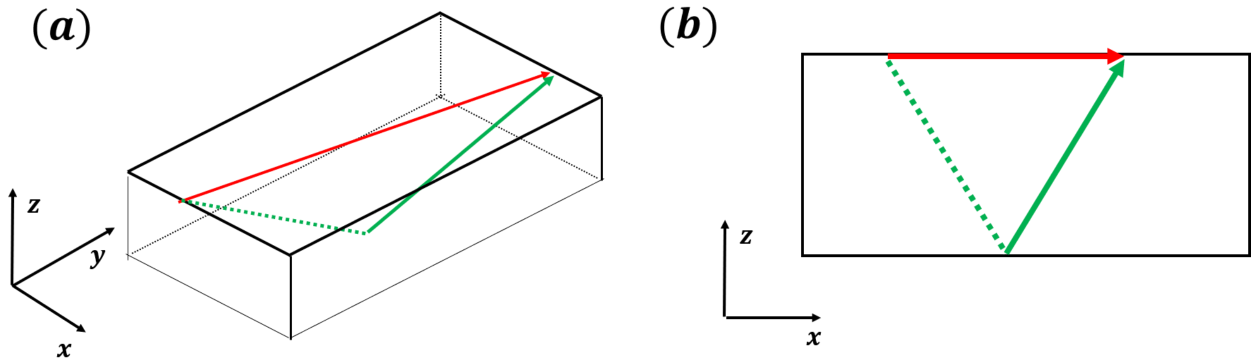

The quasiclassical trajectories of the pairing propagator inside the TI. Red lines represent surface trajectories. Green lines represent bulk trajectories that undergo a reflection. (**a**) 3D view. (**b**) Side view.

Supplementary Figure 5

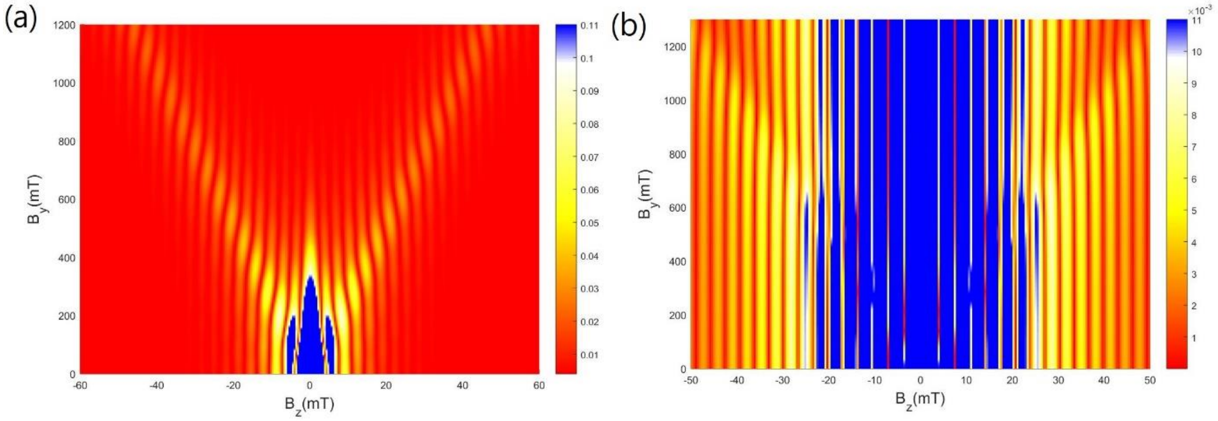

Simulations comparing the evolution of the Fraunhofer pattern for surface-dominated and bulk-dominated transport. (**a**) Surface quasiparticles will acquire an Aharonov-Bohm phase, which is reflected in an evolving Fraunhofer pattern, seen as side branches in a 2D resistance map. The simulation for device 1 from the main text is shown. (**b**) Bulk-dominated transport primarily results from trajectories with reflections, which do not pick up an Aharonov-Bohm phase. Because of this, bulk-dominated transport does not result in significant phase accumulation, which results in a Fraunhofer evolution with negligible side branches.

Supplementary Figure 6

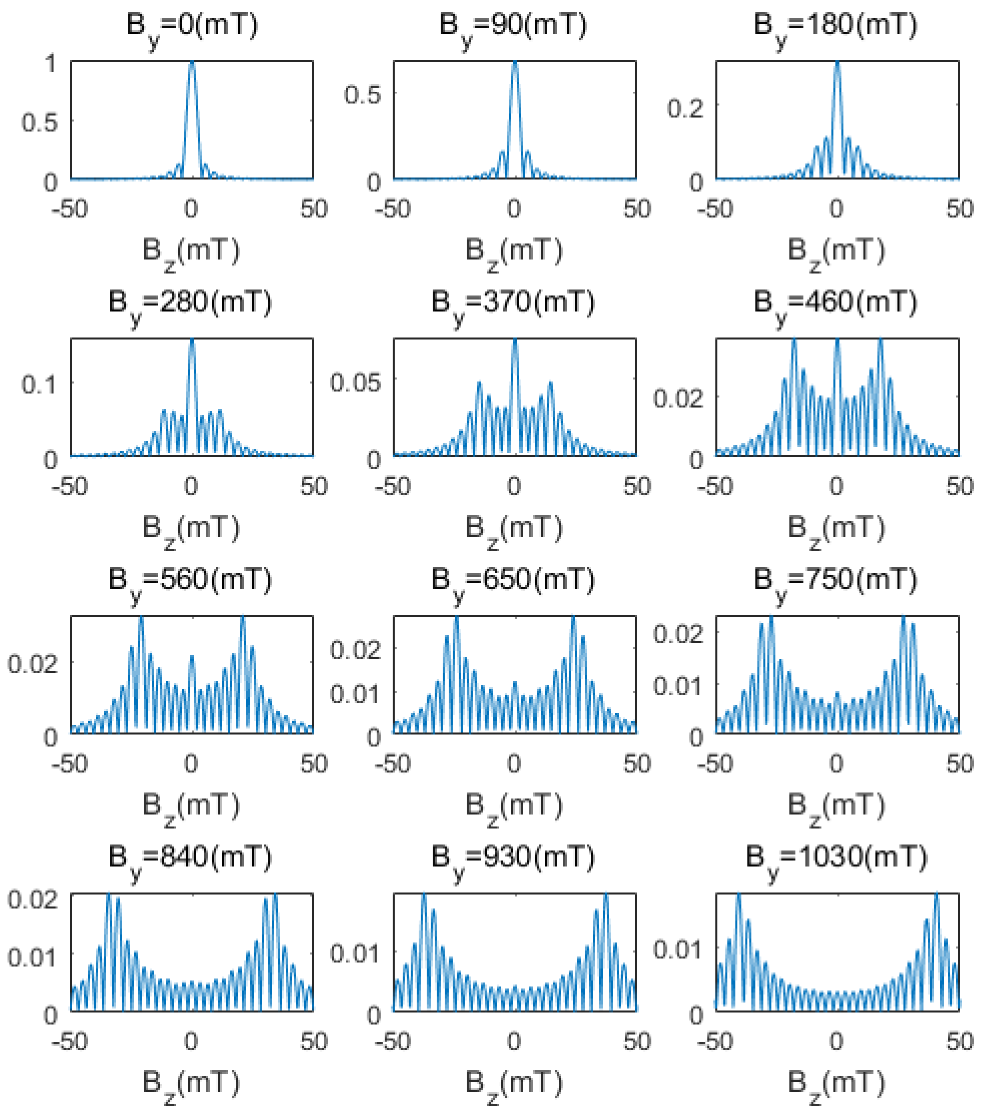

Slices of the Fraunhofer pattern for a fixed B_y. We find that the intensity of the superconductivity is transferred from the center to the sides as we increase B_y.

Supplementary Figure 7

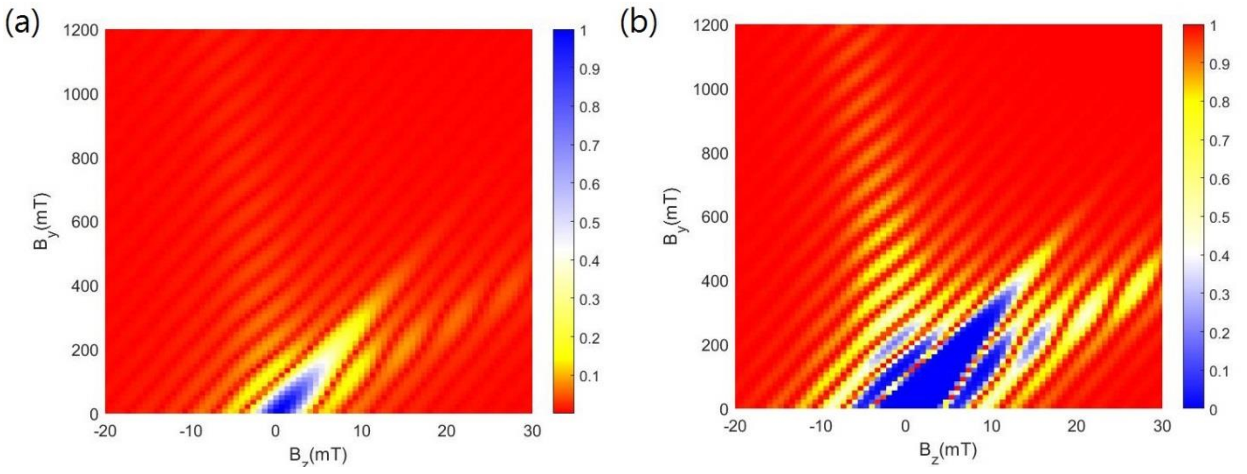

(**a**) Numerically calculated critical current map of device 1. (**b**) The corresponding normalized differential resistance calculated using Supplementary Eq. (16). If the excitation current is lower than the critical current, the differential resistance map shows a flat zero resistance region(blue).

Supplementary Table

Supplementary Table 1: Numerical parameter used in Fig. 6 in the main text.

Device number	$\alpha = \frac{W_1}{W_2}$	β	γ(B=0)
1	1.07	1/40	58
2	1.04	-1/200	58
3	1.15	0	38
4	1.02	1/100	58
5	1.00		

Supplementary Notes

Supplementary Note 1: Bulk carrier density of Bi_2Se_3 crystals

To determine the carrier density of the Bi_2Se_3 crystals used in our samples, Hall resistance R_H was measured as a function of perpendicular field B (see Supplementary Figure 1). From the Hall data, we extracted the bulk carrier density $n{\sim}5 \times 10^{17}\ \mathrm{cm}^{-3}$.

Supplementary Note 2: Effective electrode spacing *d*

For a conventional Fraunhofer pattern, the nodes occur at $B_z = \frac{n\Phi_0}{A}$, where magnetic flux quantum $\Phi_0 = \frac{h}{2e}$, A is the area of the junction, and n is an integer[1]. However, as discussed in Supplementary Reference 2, there can be variable node spacing due to a field-dependent flux focusing from the superconducting leads.

Though the overall features of our zero in-plane Fraunhofer patterns follow a conventional pattern in that they have a large central peak at $B_z = 0$ and decaying side peaks, the location of the nodes of our device Fraunhofer patterns do deviate from $B_z = \frac{n\Phi_0}{A}$ if we were to naively use area measurements obtained from an SEM image. We assume that this deviation in node position is due to flux focusing, which reduces the distance through which flux actually penetrates. The effective distance d can then be extracted from the value of the first Fraunhofer node: $d = \frac{B_{z,\text{first node}}}{W}$, where W is the width of the electrode as measured in an SEM image.

Supplementary Note 3: Discussion of slope extraction from the experimental data

As discussed in the main text, the slope of the side branches m characterizes the transfer of superconductivity intensity from the central Fraunhofer peak out to higher values of B_z as B_y is increased. In the simulations, this is defined by a line that connects the origin and the nth side lobe as n becomes large. Ideally, a slope could be similarly extracted from the data by connecting a line from the origin to the minimum of the side lobe furthest from the origin. However, the range of our experimental data is usually limited so that the minima of only 2 to 5 side lobes are lifted from the $B_y = 0$ axis. Furthermore, the experimental data can have asymmetric features (like the ones discussed in the main text or other anomalous features) that can cause the location

of individual side lobes to deviate from the side branch line drawn from the origin to a large n^{th} side lobe.

Here, we discuss how we calculate the slope of the side branches to best approximate a characteristic m for each device. After the locations of the minima of the lifted side lobes are extracted from the differential resistance map (which has been rotated so that lobe minima are vertical), a line can be drawn from the origin to each of the minima, as shown for device 1 in Supplementary Figure 2a. Supplementary Figure 2b shows the slope values $|m_n|$ (normalized by an effective electrode spacing d) for devices 1-5. Each color represents the slope of a line drawn to a different lifted side lobe, where R(L) denotes a side lobe to the right (left) of $B_z = 0$ and the number corresponds to the nth lifted side lobe. Because $|m_n|/d$ do not deviate substantially from each other for each device and all have a similar dependence on the flake thickness t, we take the average of all the $|m_n|/d$ for each device to be a good approximation to observe the relationship between slope and flake thickness. This average slope m is shown in Supplementary Figure 2a (dashed green line) and is the slope value used in the main text.

Supplementary Note 4: Details on modelling bulk transport in the Josephson junction

In this supplementary section, we model the bulk of the Josephson junction using the quasi-classical approximation. This mirrors the discussion in Supplementary Ref. 3, 4. We begin our discussion by writing down the full bulk Bogoliubov-de Gennes Hamiltonian of the 3D topological insulator, which is given as

$$H_{\text{TI}-\text{Bulk}} = \sum_{\vec{k}} \psi_{\vec{k}}^{\dagger} \begin{bmatrix} h_{\text{TI}}(\vec{k}) & 0 \\ 0 & -h_{\text{TI}}(-\vec{k})^{T} \end{bmatrix} \psi_{\vec{k}}\,, (1)$$

where $\psi_k = (c_k, c_{-k}^{\dagger})$ is the 8-dimensional Nambu spinor. The low energy normal Hamiltonian of the topological insulator is given in Ref. 4:

$$h_{\text{TI}}(k) = -E_{\text{F}} + m\Gamma_0 + \hbar v_{\text{F}} k \cdot \Gamma, \qquad (2)$$

where Γ is the Gamma matrix such that $\Gamma_0 = \tau_1\sigma_0, \Gamma_x = -\tau_3\sigma_2, \Gamma_y = \tau_3\sigma_1, \Gamma_y = \tau_2\sigma_0$. τ and σ are the Pauli matrices for the orbital and spin degrees of freedom. We can derive the Green function of the normal Hamiltonian $H_{\text{TI}-\text{Bulk}}$ by inverting the Hamiltonian:

$$G_{0,\mathrm{TI}}\left(\omega,\vec{k}\right) = \begin{bmatrix} g_{\mathrm{e,TI}}\left(\omega,\vec{k}\right) & 0 \\ 0 & g_{\mathrm{h,TI}}\left(\omega,\vec{k}\right) \end{bmatrix}, (3)$$

where the electron and hole Green functions are given as $g_{\mathrm{e,TI}}\left(i\omega,\vec{k}\right) = \frac{1}{i\omega - h_{\mathrm{TI}}(k)}$ and $g_{\mathrm{h,TI}}\left(i\omega,\vec{k}\right) = \frac{1}{i\omega + h^{*}_{\mathrm{TI}}(-k)}$.

To model bulk transport in the Josephson junction, we add the superconducting pairing on the two ends of the topological insulator in addition to the bulk Hamiltonian in Supplementary Eq. (1). Supplementary Fig. 3 shows the schematic figure of the Josephson junction. The pairing of each end can be written as

$$H_{\mathrm{pairing},1(2)} = \int dxdydz\, \psi^{\dagger}_{x,y,z} \begin{bmatrix} 0 & \Delta_{1(2)}\left(\lambda_{1(2)}\right) i\sigma_y I_2 \\ \Delta_{1(2)}\left(\lambda_{1(2)}\right)\left(i\sigma_y I_2\right)^{T} & 0 \end{bmatrix} \psi_{x,y,z}\,, (4)$$

where Δ is the pairing potential and λ_1 and λ_2 specify the location of the interface with superconductor(SC) 1 and 2 respectively. We first consider the situation where only the pairing of SC 1 is turned on ($\Delta_2 = 0$). The pairing potential of SC 1 induces the proximity effect, and the superconducting order parameter inside the topological insulator $\langle c_{\uparrow} c_{\downarrow}\rangle_1(x,y,z)$ becomes non-zero. We now perform the first order perturbation theory as a function of Δ_2. Then, the change in the energy is given as the spatial overlap of the order parameter due to SC1 and the pairing potential of SC2:

$$E_{\mathrm{Josephson}} = \int_{L+d}^{2L+d} dy \int_{0}^{W} dx\;\; \Delta_2 \langle c_{\uparrow} c_{\downarrow}\rangle_1(x,y,t). (5)$$

The Josephson current is given as $I_J = \frac{dE_{\mathrm{Josephson}}}{d\Delta\phi}$, where $\Delta\phi$ is the order parameter phase difference between SC1 and SC2.

Supplementary Note 5: Calculation of the induced order parameter and supercurrent for surface and bulk

To explicitly calculate the Josephson energy, we need to first calculate the induced order parameter $\langle c_\uparrow c_\downarrow \rangle_1(r)$. The proximity effect induced order parameter can be expressed as a function of the anomalous Green function[1], which is given as

$$\left\langle c_{\uparrow,i} c_{\downarrow,j} \right\rangle_1(\vec{r}) = \frac{1}{2\pi i} \int_{-\infty}^{0} d\omega \ \left[F^R(\omega + i\eta, \vec{r}) + \tilde{F}^R(\omega + i\eta, \vec{r})^\dagger\right], (6)$$

where $F^R(\omega + i\eta, \vec{r})$ is the retarded anomalous Green function of the topological insulator. The anomalous Green function of the topological insulator can be calculated by perturbation theory as a function of the pairing potential in SC1. We expand the full thermal Green function as a series of $H_{\text{pairing},1}$:

$$G = \frac{1}{\left(\frac{1}{G_{0,\text{TI}}} + H_{\text{pairing},1}\right)} = G_{0,\text{TI}} + G_{0,\text{TI}} H_{\text{pairing},1} G_{0,\text{TI}} + \cdots (7)$$

The first order contribution, $G_{0,\text{TI}} H_{\text{pairing},1} G_{0,\text{TI}}$, can be explicitly written as

$$G_{0,\text{TI}} H_{\text{pairing},1} G_{0,\text{TI}} = \begin{bmatrix} g_{\text{e,TI}} & 0 \\ 0 & g_{\text{h,TI}} \end{bmatrix} \begin{bmatrix} 0 & \Delta(\lambda_{1,2}) i\sigma_y I_2 \\ \Delta(\lambda_{1,2})(i\sigma_y I_2)^T & 0 \end{bmatrix} \begin{bmatrix} g_{\text{e,TI}} & 0 \\ 0 & g_{\text{h,TI}} \end{bmatrix} (8)$$

To simplify the calculation, we first consider the induced anomalous Green function due to a point source of the superconducting pairing potential located at the origin. The anomalous Green function can then be written as

$$F^1(i\omega, \vec{r}) = g_{\text{e,TI}}\,(\vec{r}) \Delta i\sigma_y I_2 \; g_{\text{h,TI}}(\vec{r}), (9)$$

$$= \frac{1}{(2\pi)^6} \int d^3k d^3k' e^{i\vec{k}\cdot\vec{r}} g_{\text{e,TI}}\,(\vec{k}) i\sigma_y I_2 e^{i\vec{k'}\cdot\vec{r}} \; g_{\text{h,TI}}(\vec{k'})$$

$$= \frac{1}{(2\pi)^6} \int d^3k d^3k'' e^{i\vec{k}\cdot\vec{r}} \frac{\sum_\lambda |\lambda_\text{e}\rangle\langle\lambda_\text{e}|}{i\omega - E_\lambda(\vec{k})} \Delta i\sigma_y I_2 e^{i\vec{k'}\cdot\vec{r}} \frac{\sum_{\lambda'} |\lambda'_\text{h}\rangle\langle\lambda'_\text{h}|}{i\omega + E_{\lambda'}(-\vec{k}')}$$

where $|\lambda_{\text{e,h}}\rangle$ is the eigenstate of the topological insulator Hamiltonian and $E_\lambda(\vec{k})$ is the corresponding energy. From here, the retarded Green function can be derived analytically. By

plugging Supplementary Eq. (9) into Supplementary Eq. (6), we get the explicit expression for the induced superconducting order parameter:

$$\langle c_{\uparrow,i} c_{\downarrow,j} \rangle_1 (\vec{r}) = \frac{-1}{2\pi i} \int_{-\infty}^{0} dE \;\; [F^R(E + i\eta, \vec{r}) + \tilde{F}^R(E + i\eta, \vec{r})^\dagger] \,, (10)$$

$$= \frac{-\Delta}{\pi} \int_{-\infty}^{0} dE \;\; \frac{1}{(2\pi)^6} \int d^3k d^3k' e^{i\vec{k} - \vec{k'} \cdot \vec{r}} \sum_\lambda |\lambda_e\rangle\langle\lambda_e| \Delta i\sigma_y I_2 \sum_{\lambda'} |\lambda'_h\rangle\langle\lambda'_h| \,\mathrm{Im}[\frac{1}{E + i\eta - E_\lambda(k)} \frac{1}{E + i\eta + E_{\lambda'}(-k')}].$$

By using the mathematical identity

$$\mathrm{Im}\left[\frac{1}{E + i\eta - E_\lambda(k)} \frac{1}{E + i\eta + E_{\lambda'}(-k')}\right]$$

$$= \frac{1}{E_\lambda(k) + E_{\lambda'}(-k')} \mathrm{Im}[\frac{1}{E + i\eta - E_\lambda(k)} - \frac{1}{E + i\eta + E_{\lambda'}(-k')}]$$

$$= -\frac{\pi}{E_{\lambda'}(-k') + E_\lambda(k)} [\delta\big(E - E_\lambda(k)\big) - \delta(E + E_{\lambda'}(-k')]$$

Supplementary Eq. (10) can be simplified as the following:

$$\langle c_{\uparrow,i} c_{\downarrow,j} \rangle_1 (\vec{r})$$
$$= \frac{\Delta}{(2\pi)^6} \int_{-\infty}^{0} dE \int_{-\infty}^{0} dE \;\; \int d^3k d^3k' e^{i\vec{k} - \vec{k'} \cdot \vec{r}} \sum_\lambda |\lambda_e\rangle\langle\lambda_e| \Delta i\sigma_y I_2 \sum_{\lambda'} |\lambda'_h\rangle\langle\lambda'_h| \frac{1}{\omega + \omega'} [\delta\big(E$$
$$- E_\lambda(k)\big)\delta(E' - E_{\lambda'}(-k')) + \delta\big(E' + E_\lambda(k)\big)\delta(E + E_{\lambda'}(-k'))]$$

$$= \Delta \int_{-\infty}^{0} \int_{-\infty}^{0} d\omega d\omega' \;\; \frac{g(E, \vec{r}) i\sigma_y I_2 g(E', \vec{r}') + g(-E, \vec{r}) i\sigma_y I_2 g(-E', \vec{r}')}{E + E'} \,, (11)$$

where $g(E, \vec{r}) = \frac{1}{(2\pi)^3} \int d^3k \sum_\lambda |\lambda\rangle\langle\lambda| \delta\big(E - E_\lambda(k)\big)$. $g(E, \vec{r})$ is similar to the spectral function of the topological insulator and is calculated in the next section.

By plugging in the normal propagators for the surface and the bulk band separately, we have derived a general expression for the induced superconducting order parameter. We can

further simplify the expression by using the quasiclassical approximation—which amounts to ignoring terms proportional to $\mathrm{e}^{\mathrm{ik_F}r}$–to look at the surface and bulk bands separately. With this approximation, the surface order parameter can be written as

$$\langle c_{\uparrow,\alpha}c_{\downarrow,\alpha}\rangle_{\mathrm{surf}}(r) = \Delta\int_{-\infty}^{0}\int_{-\infty}^{0} dEdE'\ \frac{g(E,r)i\sigma_y g(E',r) + g(-E,r)i\sigma_y g(-E',r)}{E+E'}$$

$$= \frac{\Delta k_{\mathrm{f}}}{(2\pi)^3(\hbar v_{\mathrm{f}})^2 r} e^{2i\alpha x}\int_{-\infty}^{0}\int_{-\infty}^{0} dEdE'\ \frac{4\cos(\frac{E-E'}{\hbar v_{\mathrm{F}}}r)}{E+E'}$$

$$= \frac{\Delta k_f}{(2\pi)^2\hbar v_f r^2} e^{2i\alpha x}, (12)$$

Similarly, the bulk order parameter can also be calculated:

$$\langle c_{\uparrow,\alpha}c_{\downarrow,\alpha}\rangle_{\mathrm{bulk}}(r)$$

$$= \frac{\Delta k_{\mathrm{f}}^2}{(2\pi)^4(\hbar v_{\mathrm{f}})^2 r^2}\int_{-\infty}^{0}\int_{-\infty}^{0} dEdE'\ \frac{4\sin(\frac{E+E_{\mathrm{f}}}{\hbar v_{\mathrm{f}}}r)i\sigma_y\sin(\frac{E'+E_{\mathrm{f}}}{\hbar v_{\mathrm{f}}}r) + 4\sin(\frac{-E+E_{\mathrm{f}}}{\hbar v_{\mathrm{f}}}r)i\sigma_y\sin(\frac{-E'+E_{\mathrm{f}}}{\hbar v_{\mathrm{f}}}r)}{E+E'}$$

$$= \frac{\Delta k_{\mathrm{f}}^2}{(2\pi)^4(\hbar v_{\mathrm{f}})^2 r^2}\int_{-\infty}^{0}\int_{-\infty}^{0} dEdE'\ \frac{4\cos\left(\frac{E-E'}{\hbar v_{\mathrm{f}}}r\right)}{E+E'} = \frac{\Delta k_{\mathrm{f}}^2}{(2\pi)^3\hbar v_{\mathrm{f}} r^3}, (13)$$

Thus, we have derived the induced superconducting order parameter for the surface and bulk states. It is important to note that the bulk order parameter decays as a function of $1/r^3$ while the surface order parameter decays as $1/r^2$. Furthermore, the surface order parameter oscillates as $e^{2i\alpha x}$, indicating the presence of the ZME. In contrast, the bulk order parameter is uniform since the bulk Fermi surface remains centered at Γ point.

We can now calculate the surface and bulk Josephson currents by plugging Supplementary Eqs. (12) and (13) into

$$E_{\text{Josephson}} = \int_{L+d}^{2L+d} dy \int_0^W dx\ \Delta_2 \langle c_\uparrow c_\downarrow \rangle_1 (x, y, t).$$

The Josephson current/coupling between the superconductors is given as

$$I_{\text{surf}} \approx \int_0^W dx_1 dx_2 \int_0^\infty dy_1 \frac{\Delta k_{\text{f}}}{(2\pi)^2 \hbar v_{\text{f}}((d+y_1)^2 + (x_1 - x_2)^2)} \sin\big(\Delta\phi + 2\alpha(x_1 - x_2)\big) \qquad (14)$$

$$I_{\text{bulk}} \approx \int_0^W dx_1 dx_2 \int_0^\infty dy_1 \int_0^L dy_2 \frac{\Delta k_{\text{f}}^2}{(2\pi)^3 \hbar v_{\text{f}}((d+y_1+y_2)^2 + (x_1 - x_2)^2)^{\frac{3}{2}}} \sin(\Delta\phi). (15)$$

The power of the denominators in Supplementary Eq. (14) and (15) reflects how the supercurrent decays inside the junction: the bulk Josephson coupling decays faster than the surface coupling as the distance between the superconductor increases.

Supplementary Note 6: Evolution of the Fraunhofer pattern due to the bulk

As discussed in the previous section, the trajectories of the bulk electrons can be characterized as trajectories with reflections and trajectories without reflections. Furthermore, we can also estimate the amount of supercurrent originating from the reflected bulk electrons and non-reflected bulk electrons. According to Supplementary Eq. (13), the bulk proximity effect decays as a function of $1/r^3$, where r is the length of the trajectory. Therefore, $F_n(i\omega, \mathrm{r})$—where F is roughly proportional to the supercurrent—with n reflections scales as $1/(d^2 + (2nt)^2)^{3/2}$. The ratio between the reflected and non-reflected supercurrent can then be written as

$$\frac{I_{\text{with reflections}}}{I_{\text{no reflections}}} \sim \frac{|\sum_{n=1}^{\infty} F_n|}{|F_0|} = \frac{\sum_{n=1}^{\infty} \frac{1}{(d^2+(2nt)^2)^{\frac{3}{2}}}}{\frac{1}{d^3}} = \sum_{n=1}^{\infty} \frac{1}{\left(1+\left(\frac{2nt}{d}\right)^2\right)^{\frac{3}{2}}}.$$

Our Josephson junctions have a narrow geometry, where $\frac{t}{d} \lesssim \frac{1}{10}$. In this regime, the number of possible reflected trajectories is larger than the number of non-reflected trajectories. For example, for device 1, $\frac{t}{d} = \frac{9\text{ nm}}{140\text{ nm}}$, so $\frac{I_{\text{with reflections}}}{I_{\text{no reflections}}} \sim \frac{|\sum F_n|}{|F_0|} \sim 7.27$. Therefore, in our devices, the reflected bulk supercurrent dominates over the non-reflected bulk supercurrent.

As discussed in the methods section of the main text, the electrons with reflected trajectories do not gain a phase due to the FME. Therefore, because most of the bulk current comes from reflected trajectories, the bulk acquires a negligible FME phase accumulation and there would be little phase accumulation in bulk-dominated transport. As a result, the Fraunhofer pattern would not substantially evolve when an in-plane field B_y is applied; Supplementary figure 5b shows a simulation for the case of bulk-dominated transport. This contrasts starkly with what we observed in our experiments, which more closely matches simulations that take into account phase accumulation due to surface-dominated transport (Supplementary figure 5a).

Supplementary Note 7: Details of the numerical methods

To compare simulations of critical current with the experimental data, we map the critical current map as a function of B_y and B_z to a differential resistance map. In order to transform the Fraunhofer pattern into the differential resistance map, we model an effective thermal noise using Ambegaokar-Halperin (AH) theory, which is given as

$$V = \frac{4\pi}{\gamma}\left\{(e^{\pi\gamma x}-1)^{-1}\left[\int_0^{2\pi} d\theta f(\theta)\right]\left[\int_0^{2\pi} d\theta \frac{1}{f(\theta)}\right] + \left[\int_0^{2\pi}\int_\theta^{2\pi} d\theta d\theta' \frac{f(\theta)}{f(\theta')}\right]\right\}^{-1} \quad (16)$$

Here V and x are normalized voltage and current, respectively. $f(\theta) = e^{\frac{1}{2}\gamma(x\theta+cos\theta)}$. γ is an effective dimensionless parameter representing thermal fluctuations, which is typically given as $\gamma = \frac{\hbar I_C}{eK_bT}$. By fitting d$V$/d$I$ calculated using Supplementary Eq. (16) to the measured I-V characteristics of the experiment, we extract the effective value of the thermal fluctuations. We

find that the extracted temperature from the AH theory does not necessarily match the base temperature of the experiment ($\mathrm{T} = 25\ \mathrm{mK}$), indicating a possible increase in the temperature inside the device or fluctuations due to noise, but we also point out that it has been reported that the AH theory has shown quantitative discrepancies in underdamped junctions. Therefore, we instead treat γ as a fitting parameter to account for the effective fluctuations in the device.

Supplementary Figure 7 shows the comparison between the critical current map of the evolution of the Fraunhofer pattern and the corresponding differential resistance map. When the critical current is larger than the excitation current, the differential resistance becomes negligible, corresponding to the flat blue region. If the excitation current is higher than the critical current, it is seen as red resistive region in the differential resistance map. Supplementary Table 1 summarizes the numerical parameters used to generate Fig. 6 in the main text.

Supplementary References

1. Tinkham, M. *Introduction to Superconductivity* (Dover Publications Inc., 2013).
2. Suominen, H. J. *et al.* Anomalous Fraunhofer interference in epitaxial superconductor-semiconductor Josephson junctions. *Phys. Rev. B* **95**, 035307 (2017).
3. Hart, S. *et al.* Controlled finite momentum pairing and spatially varying order parameter in proximitized HgTe quantum wells. *Nat. Phys.* **13**, 87-93 (2017).
4. Mohammadkhani, G., Zareyan, M., & Blanter, Ya. M. Magnetic interference pattern in planar SNS Josephson junctions, *Phys. Rev. B* **77**, 014520 (2008).
